# Serum exosomes from young rats improve the reduced osteogenic differentiation of BMSCs in aged rats with osteoporosis after fatigue loading in vivo

**DOI:** 10.1186/s13287-021-02449-9

**Published:** 2021-07-27

**Authors:** Jingqiong Xun, Chan Li, Meilu Liu, Yueming Mei, Qiongfei Zhou, Bo Wu, Fen Xie, Yuling Liu, Ruchun Dai

**Affiliations:** 1 National Clinical Research Center for Metabolic Diseases, Institute of Metabolism and Endocrinology, Central South University, Hunan Provincial Key Laboratory of Metabolic Bone Diseases, and Department of Metabolism and Endocrinology, The Second Xiangya Hospital of Central South University, Changsha, Hunan China; 2 Department of Endocrinology, Affiliated Hospital of Guilin Medical University, Guilin, Guangxi China; 3 Department of Endocrinology, Xiangtan Central Hospital, Xiangtan, Hunan China

**Keywords:** Bone microdamage, Bone mesenchymal stem cells, Exosomes, Ageing, Osteogenic differentiation, Osteoporotic fracture, MicroRNA

## Abstract

**Background:**

Osteoporosis is a major public health concern for the elderly population and is characterized by fatigue load resulting in bone microdamage. The ability of bone mesenchymal stem cells (BMSCs) to repair bone microdamage diminishes with age, and the accumulation of bone microdamage increases the risk of osteoporotic fracture. There is a lack of effective means to promote the repair of bone microdamage in aged patients with osteoporosis. Exosomes have been shown to be related to the osteogenic differentiation of BMSCs. Here, we aimed to evaluate the changes in the osteogenic differentiation capacity of BMSCs in aged osteoporotic rats after fatigue loading and the treatment potential of serum exosomes from young rats.

**Methods:**

The tibias of six aged osteoporotic rats were subjected to fatigue loading in vivo for 2 weeks, and the bone microdamage, microstructures, and mechanical properties were assessed. Subsequently, BMSCs were extracted to evaluate their proliferation and osteogenic differentiation abilities. In addition, the BMSCs of aged osteoporotic rats after fatigue loading were treated with serum exosomes from young rats under osteogenic induction conditions, and the expression of osteogenic-related miRNAs was quantified. The osteogenetic effects of miRNA-19b-3p in exosomes and the possible target protein PTEN was detected.

**Results:**

Obvious bone microdamage at the fatigue load stress point, the bone microstructure and biomechanical properties were not obviously changed. A decreased osteogenic differentiation ability of BMSCs was observed after fatigue loading, while serum exosomes from young rats highly expressing miRNA-19b-3p improved the decreased osteogenic differentiation ability of BMSCs. Transfection with miRNA-19b-3p mimic could promote osteoblastic differentiation of BMSCs and decreased the expression of *PTEN*. After transfection of miRNA-19b-3p inhibitor, the promotional effect of exosomes on bone differentiation was weakened. Treatment with transfected exosomes increased the expression of *PTEN*.

**Conclusion:**

Serum exosomes derived from young rats can improve the decreased osteogenic differentiation ability of BMSCs in aged rats with osteoporosis after fatigue loading and can provide a new treatment strategy for the repair of bone microdamage and prevention of fractures.

**Supplementary Information:**

The online version contains supplementary material available at 10.1186/s13287-021-02449-9.

## Background

The incidence of osteoporosis in the elderly population is high, and the number of patients with osteoporosis increases as the social population ages. The occurrence of osteoporotic fractures is closely related to bone strength, which is affected by not only bone mass or bone density (BMD) but also, importantly, bone quality [1] throughout the hierarchical levels of bone structure [2–4]. Microdamage accumulation is an important determinant of bone quality [5] that increases with ageing in a linear microcrack form and is considered to contribute to bone fragility, resulting in an increased risk of fracture, particularly in elderly individuals [6]. However, the mechanism underlying the accumulation and repair of microdamage during the occurrence and development of fractures remains unclear, and therapeutic strategies to improve the repair of bone microdamage in aged individuals with osteoporosis and to reduce the risk of fracture remain limited. Therefore, an in-depth understanding of microdamage repair before fracture occurrence and development in age-related osteoporosis can provide effective approaches for the early treatment and prevention of osteoporotic fractures.

The function of bone mesenchymal stem cells (BMSCs) is crucial to the outcome of bone damage repair. After bone tissue injury, osteocytes sense mechanical changes and microdamage and initiate bone resorption, which is mediated by osteoclasts, and new bone generation, which is mediated by osteoblasts derived from BMSCs. Osteoclasts and osteoblasts jointly promote bone remodelling [7–9] and microdamage repair [10–12]. With increasing age, the number of BMSCs remains the same, whereas that of mature osteoblasts decreases [13], suggesting that the osteogenic differentiation ability of BMSCs is impaired over time, leading to the failure of timely microdamage repair, bone loss, and an increased incidence of fractures [14, 15]. Improving the osteogenic differentiation of BMSCs in aged subjects with osteoporosis after fatigue loading may provide a new treatment strategy for bone microdamage repair and fracture prevention.

Exposure to youthful circulation by heterochronic parabiosis has been shown to reverse the aged fracture repair phenotype and diminish the osteoblastic differentiation capacity in old mice [15]. Extracellular vesicles (EVs) mainly comprise exosomes and microvesicles and have functional effects within their respective tissues; however, evidence shows that they are also shed into the circulation and can affect distant tissues. Injection of some EVs into the hypothalamic third ventricle was shown to ameliorate some age-associated phenotypes in mice [16], suggesting that EVs are mediators of circulating communication. Circulating EVs in young organisms can promote longevity and health [17]. One study reported that the osteo-inductive potential of human plasma-derived EVs decreases with age, contributing to reduced bone formation and increased fracture risk [18]. Exosomes, lipid bilayer-enclosed vesicles with a diameter ranging between 30 and 100 nm, are produced when the membranes of intracellular multivesicular bodies fuse with the cytoplasmic membrane and can be released by many cell types through exocytosis [19] into the extracellular environment or biological fluids. Exosomes have diverse functions, such as remodelling the extracellular matrix and transmitting signals and molecules such as microRNAs (miRNAs), proteins, and lipids [20, 21] to other cells. Exosomes contribute to an array of normal physiological processes (e.g., development, ageing, metabolic regulation, exercise, stress, and interactions), many non-infectious diseases, and infectious diseases [22]. Thus, we speculate that exosomes play an important role as an important bioactive medium in circulating blood. We hypothesize that young serum exosomes promote the osteogenic differentiation ability of BMSCs to repair bone microdamage.

miRNAs are short (~ 22 nucleotides) noncoding RNAs targeting specific mRNAs to regulate posttranscriptional gene expression [23] and are key regulators of BMSC osteogenic differentiation by directly targeting mRNAs related to osteogenic differentiation. Previous studies have indicated the presence of differentially expressed circulating miRNAs in patients with fractures [24], suggesting that miRNAs in circulation are closely related to bone remodelling and involved in bone damage repair.

In this study, we observed the effect of fatigue loading on the osteogenic differentiation ability of BMSCs in aged osteoporotic rats. We hypothesized that the serum exosomes of young rats would improve the osteogenic differentiation ability of BMSCs by encapsulating miRNAs. Furthermore, these results can provide ideas for a new treatment strategy for bone microdamage repair and fracture prevention.

## Methods

### Experimental animals

Female clean-grade Sprague-Dawley (SD) rats (1 and 7 months old) were provided by the Laboratory Animal Center of the Second Xiangya Hospital of Central South University. The rats were housed at 25 ± 2 °C with 45–55% humidity and a light/dark duration of 12/12 h. All animal procedures were approved by the Animal Care Committee of the Second Xiangya Hospital of Central South University. All animal experiments were officially approved by the Ethics Committee of the Second Xiangya Hospital, Central South University. One-month-old rats were used for the extraction of serum exosomes.

### Ovariectomy and bone mineral density test

Bilateral variations in 7 months old rats were removed to establish the ovariectomy (OVX) rat model. Briefly, rats were anaesthetized with 3% pentobarbital sodium (1 mL/kg). After fallopian tube ligation, the bilateral ovaries were clamped and removed, and the skin was sutured. OVX rats were raised until 22 months of age to establish aged rats with bone microdamage by fatigue loading. The BMD area was assessed by dual-energy X-ray absorptiometry (DXA) using a Lunar DPX densitometer (GE Healthcare, Lunar Corporation, Madison, WI, USA) before OVX [pre-OVX (7 months old)], 3 weeks after OVX [post-OVX (7 months old)], and before fatigue loading [post-OVX (22 months old)].

### Three-point bending test

A three-point bending failure test was performed on the right tibias of 22-month-old female rats before mechanical loading using the electronic universal testing machine WDW3100 (Changchun Testing Machine Institute, Changchun, China). The fulcrum span (L) was 16 mm, and the load was applied vertically at the centre (the femur was at a 90-degree angle to the load) at 10 mm/min until the tibia broke. The load–deformation curve was drawn using matching image analysis software, and data were analysed to determine the ultimate tibial load value (Fmax), maximum bending strength, stiffness constant K, and elastic modulus. According to the Fmax values of the tibias of aged rats with osteoporosis [post-OVX (22 months old)], the value of the force required for mechanical loading was determined. Aged osteoporotic rats were randomly divided into two groups: the unloading and loading groups (*n* = 6 each). After mechanical loading, the tibial biomechanical properties, including the maximum bending strength, Fmax, maximum bending strength, and elastic modulus, were analysed and compared between aged osteoporotic rats in the unloading and loading groups.

### Fatigue load

Aged osteoporosis rats were randomly divided into two groups: the unloading and loading groups (*n* = 6 each). Rats in the loading group were anaesthetized. A self-developed machine for electronically measuring fatigue damage (fixture SL-2000, China patent number: ZL00225310.0) was used to complete the four-point bending fatigue test as described previously (Sup. Fig. 1A, B). Our previous studies found that the force of microdamage modelling was 40–60% of the ultimate load. An initial rate of 1 mm/min and the force value control of the rat tibial four-point bending test were set according to the ultimate load value of the tibia measured by the three-point bending test (Table 1). Anaesthetized rats of the loading group were subjected to cyclic mechanical loading at 45 N peak force and 35 N trough force loads (sinusoidal wave: 4 Hz, 10000 cycles). The loads were applied to both tibial parts every other day for 2 weeks to cause bone microdamage, and the force-bearing point (Sup. Fig. 1C) was marked. After the loading experiment, the tibias were harvested. The microdamage in the left tibial bones of rats in the two groups was observed and compared, whereas the right tibia was analysed to observe the microstructures and evaluate the biomechanical properties.

**Table 1:** Tibial load in aged ovariectomized rats(*n*=4,‾X±SD)

The degree of load	Load force value (N)
Ultimate load (Fmax)	85.618±12.655
40% ultimate load	34.247±5.062
50% ultimate load	42.809±6.327
60% ultimate load	51.371±7.593

### Bone microdamage evaluation

Bone microdamage was detected and evaluated using basic magenta staining and optical microscopy. Tibias were dehydrated and stained in ascending alcohols containing 1% basic fuchsin, subjected to hyalinization and dimethylbenzene, and embedded in polymethylmethacrylate until polymerization was complete. Thick sections (80–100 μm) were cut transversely and sequentially using a diamond saw at the force-bearing points and observed using a Leica DMLA polarized light microscope (Leica Corporation, Wetzla, Germany). Images of areas with microdamage were captured, and Leica Qwin (Leica Corporation, Wetzla, Germany) was used to calculate the values of the microdamage parameters, such as the average microcrack length (Cr. Le), number of microcracks (Cr. N), microcrack surface density (Cr. S. Dn), and microcrack density (Cr. Dn). Data from four sections of each tibia were collected.

### Microcomputed tomography (μCT) analysis

Right tibias were scanned using SkyScan-1176 microcomputed tomography (μCT) (Bruker micro-CT, Belgium). The scanner voltage and current were set to 80 kV and 278 μA, respectively, with a resolution of 18 μm per pixel. The NRecon (v1.6) and CTan (v1.13) software programmes were used for image reconstruction and bone analysis, respectively. The X position was assigned based on the load point (Supp. Fig. 1D), and a region encompassing a minimum distance of 3–5 mm and a maximum distance of 8–10 mm from the X position was selected as the region of interest (ROI) for analysis. The tibial mineral density, volume, volume percentage, and cortical thickness were assessed.

### BMSC isolation and culture

BMSCs were isolated from rat tibias immediately after euthanization. Briefly, the remaining muscles on the bone surface were removed, and the epiphysis at both ends was cut to expose the bone marrow cavity. The bone marrow cavity was rinsed with low-glucose Dulbecco’s modified Eagle’s medium (DMEM), and bone marrow was collected and centrifuged at 1200 rpm for 5 min at room temperature. After centrifugation, the pellets were cultured in low-glucose DMEM supplemented with 10% foetal bovine serum (FBS) and 1% penicillin and streptomycin. The cells were seeded at a density of 1 × 10^6^ cells/mL and cultured in a humidified incubator at 37 °C with 5% CO_2_.

### BMSC characterization

BMSCs passaged three to four times with a uniform morphological appearance as fibroblast-like long spindles in an ordered arrangement were used. BMSC surface marker expression was analysed by incubating the samples with CD45-FITC (1:1000, eBioscience, Thermo), CD34-FITC (1:400, eBioscience, Thermo), and CD29-PE (1:100, eBioscience, Thermo) antibodies for 30 min at 4 °C in the dark, followed by flow cytometry on a FACS Calibur instrument (Becton Dickinson). The expression of surface antigens on BMSCs was analysed using FlowJo software. BMSCs were induced by osteogenic and adipogenic medium. The osteogenic medium was supplemented with 10% FBS, 10 μmol/L β-glycerophosphate, 0.1 μmol/L dexamethasone, 50 μg/mL ascorbate, and 10 μmol/L glutamine to detect the changes in osteogenic differentiation capacity. The adipogenic medium was supplemented with 1000 nmol/L dexamethasone, 0.5 mmol/L 3-isobuthyl-1-methylxanthine (IBMX) solution (Solarbio), and 5 μg/ml insulin (Sigma). The medium was replaced every three days. After osteogenesis induction for 14 days, Western blot (WB) was used to detect osteogenesis-related proteins (collagen I and RUNX2) in BMSCs, and quantitative real-time PCR (RT-qPCR) was used to evaluate the expression of osteogenesis-related genes (*ALP*, *collagen I*, *RUNX2*, and *OCN*). Alizarin Red and Oil Red O staining were used to identify calcium nodules and lipid droplets, respectively. The results were observed via microscopy.

### Exosome isolation and characterization

After the anaesthetization of 1-month-old rats, blood was collected through the abdominal aorta, left at room temperature for 1 h and centrifuged at 3000×*g* for 10 min. The supernatant was collected, and exosome extraction was performed using an SBI ExoQuick according to the manufacturer’s instructions with some modifications. Briefly, the serum was centrifuged at 3000×*g* for 30 min, then diluted with PBS (1:1) and centrifuged at 10,000×*g* for 40 min at 4 °C; the supernatant was then transferred to an ultrafiltration tube (Millipore Amicon Ultra 10 kd) and centrifuged at 3000×*g* for 40 min at 4 °C. The filtrate was added to the ExoQuick exosome precipitation solution at a ratio of 250:63, and the solution was incubated for 30 min and then centrifuged at 1500×*g* for 30 min. The supernatant was removed, and the remaining sample was centrifuged at 1500×*g* for 5 min, followed by discarding of the residual supernatant. The obtained pellet was resuspended in PBS and stored at 4 °C or − 20 °C. The exosomes were morphologically analysed by transmission electron microscopy (Tecnai G2 Spirit Twin, FEI), and their diameters were determined using the Nanosight 2000 instrument (Particle Metrix). Exosomal marker proteins, including CD9, CD81, and TSG101, were analysed by WB.

### BMSCs and exosome transfection

The miRNA-19b-3p mimic and negative control, miRNA-19b-3p inhibitor and negative control were synthesized (RiboBio, Guangzhou, China). The miRNA-19b-3p mimic or negative control were transfected using the HiPerFect transfection reagent (Qiagen). Briefly, 12 μL of HiPerFect reagent was added to diluted miRNA-19b-3p mimic or negative control in medium without serum, respectively. Samples were incubated at room temperature for 10 min and added onto the cells cultured in a medium without FBS. After 8 h, the medium was replaced with an osteogenic differentiation medium. The miRNA-19b-3p inhibitor and negative control were transfected used the Exosome Transfection Kit (System Biosciences, California, USA). Briefly, 10 μL of the Exo-Fect solution, 20 μL of the miR-19b-3p inhibitor or negative control (3 μM), 60 μL of purified FBS-Exos and 60 μL of PBS were mixed in a clean 1.5 mL tube, incubated on a shaker at 37 °C for 10 min and then immediately placed on ice. After the addition of 30 μL of ExoQuick-TC reagent to stop the reaction, the mixture was placed at 4 °C for 30 min and then centrifuged at 14,000 rpm for 3 min. The supernatant was removed, and the exosome pellet was resuspended in PBS for use.

### Alkaline phosphatase (ALP) and Alizarin Red staining

BMSCs were washed three times with PBS and fixed with 4% paraformaldehyde for 30 min.

Alkaline phosphatase staining was performed using the BCIP/NBT Alkaline Phosphatase Color Development Kit (Beyotime) according to the manufacturer’s instructions. After incubation for 12 h, they were determined using a scanner and observed microscopically. BMSCs were fixed in 4% paraformaldehyde for 30 min and stained by Alizarin Red for 5 min. The orange and red positions were recognized as calcium deposition.

### Uptake of exosomes by BMSCs

For the exosome internalization experiments, the purified exosomes were labelled with a PKH67 Green Fluorescent Cell Linker Kit according to the manufacturer's instructions. BMSCs were incubated with PKH67-labelled exosomes for 24 h in the dark and fixed with 4% paraformaldehyde for 15 min. The nuclei were stained with DAPI, and the STExo uptake signals were observed using laser confocal microscopy (LSM 780, AxioObserver, Zeiss).

### Cell growth assay

The growth and viability of BMSCs were determined using the Cell Counting Kit-8 (CCK8) assay. BMSCs (2000 cells per well) were seeded into 96-well tissue culture plates and washed twice with PBS, after which the absorbance at 450 nm was measured. The cells counts were determined, and the CCK8 assay was repeated three times.

### Western blot analysis

WB was used to detect the levels of β-actin, GAPDH, collagen I, RUNX2, PTEN, CD9, CD81, and TSG101 as previously described. Proteins were separated using sodium dodecyl sulphate-polyacrylamide gel electrophoresis and transferred onto polyvinylidene fluoride membranes. After blocking with 5% skim milk, the membranes were incubated with β-actin, GAPDH (1:2000 dilution, Proteintech), Collagen I (1:1000, Proteintech), RUNX2 (1:2000, Abcam), PTEN (1:2000, Proteintech), CD9 (1:1000, Abcam), CD81 (1:2000, Abcam), and TSG101 (1:500, Abcam) antibodies overnight at 4 °C. The membranes were then washed with PBS three times for 10 min each and incubated with an appropriate secondary antibody (1:4000, Proteintech) in 2% skim milk for 1 h. Bands were processed using an enhanced chemiluminescence (ECL) kit, visualized using an Amersham Imager 600 imaging system (GE), and analysed by densitometry.

### Quantitative real-time PCR

Total RNA was extracted from BMSCs and exosomes using TRIzol reagent (Invitrogen), and the RNA quality was evaluated using a Nanodrop 2000 spectrophotometer. The RNA was reverse-transcribed into cDNA using a Prime script RT reagent Kit (Takara) and a Mix-XTM miRNA First Strand Synthesis Kit (TaKaRa). RT-qPCR was performed on a Light Cycler 96 system (Roche) using TB Green Premix Ex Taq (TaKaRa) protocols. Relative changes in gene expression were assessed using the 2^–ΔΔCt^ method, and GAPDH served as the internal control. The primer sequences are listed in Table 2.

**Table 2:** Sequences of primers

Gene name	Primer sequence
Runx2-F	5′-GACTGTGGTTACCGTCATGGC-3′
Runx2-R	5′-ACTTGGTTTTTCATAACAGCGGA-3′
ALP-F	5′- TGTTGGTCCTGCTGGCAAGAATG-3′
ALP-R	5′-CAGGCACAGTGGTCAAGGTTGG -3′
CollagenI-F	5′- TGTTGGTCCTGCTGGCAAGAATG -3′
CollagenI-R	5′-GTCACCTTGTTCGCCTGTCTCAC -3′
PTEN-F	5′-TTGAAGACCATAACCCACCACAGC -3′
PTEN-R	5′-CATTACACCAGTCCGTCCTTTCCC -3′
GAPDH-F	5′-AGCCCAAGATGCCCTTCAGT-3′
GAPDH-R	5′-CCGTGTTCCTACCCCCAATG-3′
miR-9a-5p	5′-CGCCGTCTTTGGTTATCTAGCTGTATG-3′
miR-19b-3p	5′-CCGTGTGCAAATCCATGCAAAACTGA-3′
miR-20a-5p	5′-CGCCTAAAGTGCTTATAGTGCAGGTAG-3′
miR-335-5p	5′-CGCGCTCAAGAGCAATAACGAAAAATG-3′
miR-433-3p	5′-CGCCGTCTTTGGTTATCTAGCTGTATG-3′
miR-21-5p	5′-CGCCGTAGCTTATCAGACTGATGTTGA-3′
miR-181a	5′-AACATTCAACGCTGTCGGTGAGT-3′
miR-29b	5′-CGCCTAGCACCATTTGAAATCAGTGTT-3′
miR-199a-5p	5′-GCCCAGTGTTCAGACTACCTGTTC-3′
U6-F	5′- GGAACGATACAGAGAAGATTAGC-3′
U6-R	5′-TGGAACGCTTCACGAATTTGCG-3′

### Statistical analysis

Data are presented as the mean ± SD or SEM. Data analysis was performed using Statistical Product and Service Solutions (SPSS) software (version 19.0). Two-group comparisons were performed using *t*-tests (and nonparametric tests), whereas multi-group comparisons were performed using one-way analysis of variance (ANOVA). *P* < 0.05 was considered statistically significant. All experiments were repeated at least three times.

## Results

### Fatigue loading caused bone microdamage but did not alter the bone microstructural or biomechanical properties in aged osteoporotic rats

**Fig. 1:**
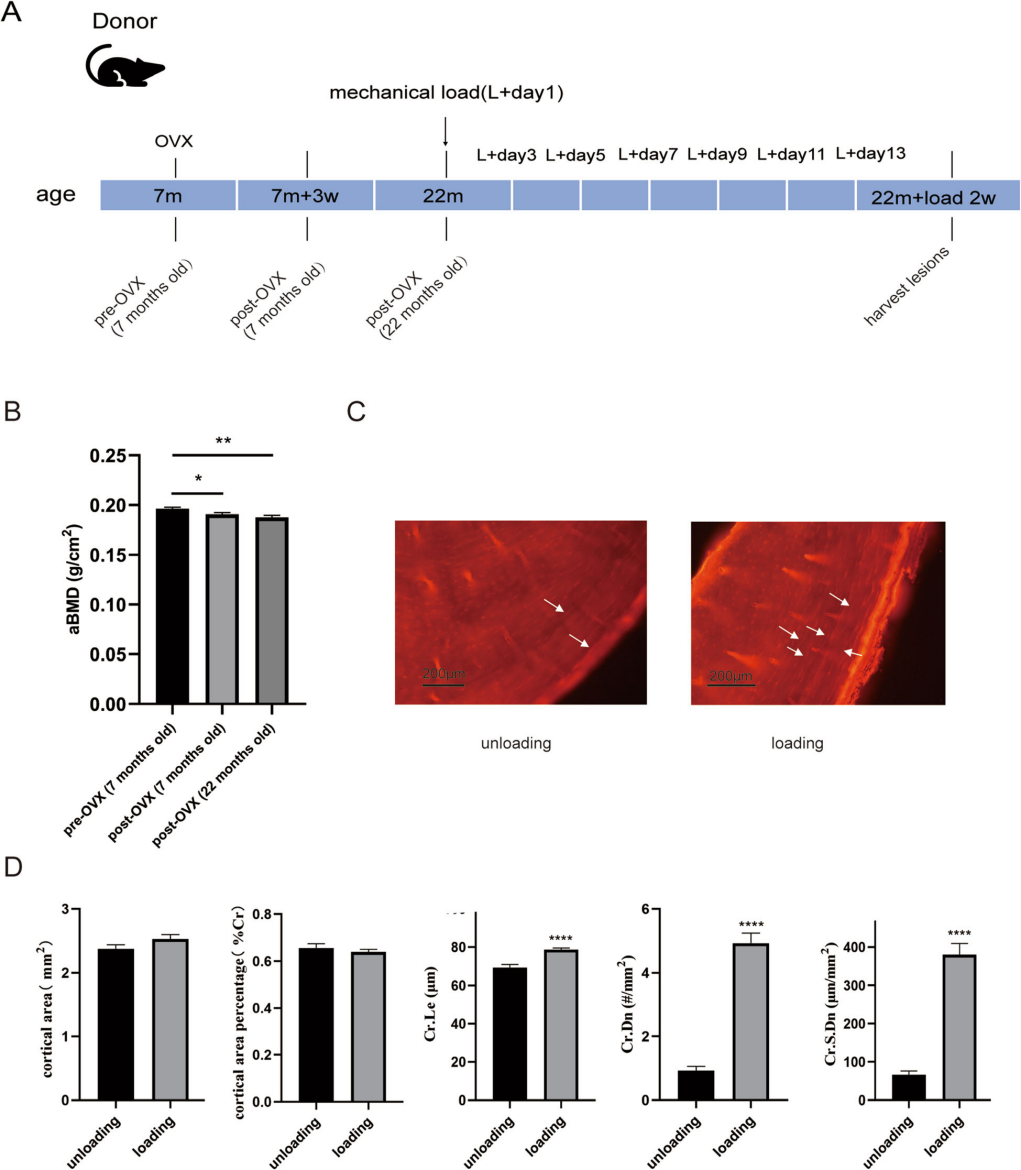
Bone mineral densities of aged osteoporotic rats with bone microdamage and bone microdamage after mechanical loading. **A** Experimental diagram of the induction of bone microdamage in aged osteoporotic rats. **B** BMD areas of the vertebral bodies of SD females before and after OVX as measured using DAX (pre-OVX (7 months old) and post-OVX (7 months old), *n* = 19; post-ovx (22 months old), *n* = 13). **C** Bone microdamage as observed using a fluorescence microscope (white arrow). Scale bar: 200 μm. **D** Microdamage parameters, including the cortical bone area, proportion of cortical bone area, microcrack length (Cr. Le), microcrack density (Cr. Dn), and microcrack surface density (Cr. S. Dn) (*n* = 4). The data are expressed as the mean ± SEM. *P < 0.05, **P < 0.01, ****P < 0.0001

Figure 1B shows that the BMD of post-OVX (7 months old) rats and post-OVX (22 months old) rats was significantly lower than that of pre-OVX (7 months old) rats (*P* < 0.05, *P* < 0.01), demonstrating that the osteoporosis model was established successfully. However, the BMD of post-OVX 22 months old) rats was not significantly reduced compared with that of post-OVX (7 months old) rats (*P >* 0.05) (Fig. 1B). The ultimate load value of the post-OVX (22 months old) rat tibia measured using a three-point bending test was 85.618 ± 12.655 N (*n* = 4). The tibias of the rats in the loading group had more microdamage, as shown by the white arrow (Fig. 1C). The cortical area percentage was not significantly different between the loading and unloading groups (*P >* 0.05). The average microcrack length (Cr. Le), microcrack density (Cr. Dn), and microcrack surface density (Cr. S. Dn) of the tibias in the loading group were higher than those in the unloading group (*P* < 0.001) (Fig. 1D).

**Fig. 2:**
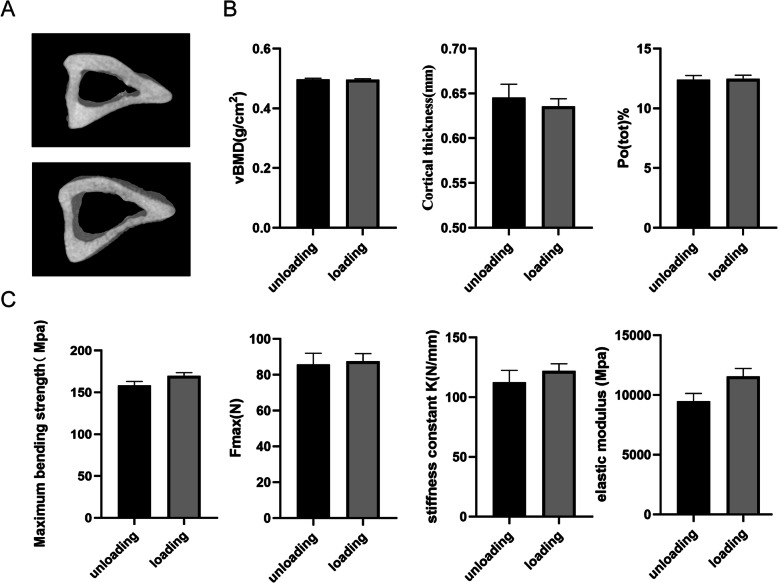
Comparison of the tibial microstructures and biomechanical parameters of rats in the loading and unloading groups. **A** Three-dimensional reconstruction of the bone microstructure. **B** Volume bone mineral density and tibial bone microstructure parameters: cortical thickness and total porosity percentage, Potot. **C** Biomechanical parameters: maximum bending strength, ultimate tibial load value (Fmax), stiffness constant K, and elastic modulus. The data are expressed as the mean ± SEM. (*n* = 4)

Fatigue loading caused bone microdamage, but the bone microstructural and biomechanical properties of the rats were not changed by fatigue loading. The three-dimensional reconstructions of the bone microstructure (Fig. 2A) and the BMD volume (vBMD) and bone microstructure parameters of the tibia, such as cortical thickness and total porosity percentage (Po_tot_), were not significantly different between the loading and unloading groups (*P >* 0.05) (Fig. 2B). There were also no significant differences in the biomechanical indicators, including the maximum bending strength, Fmax, stiffness constant K, and elastic modulus, between the two groups (*P >* 0.05) (Fig. 2C).

### The proliferation and osteogenic differentiation abilities of BMSCs were reduced in the loading group

**Fig. 3.**
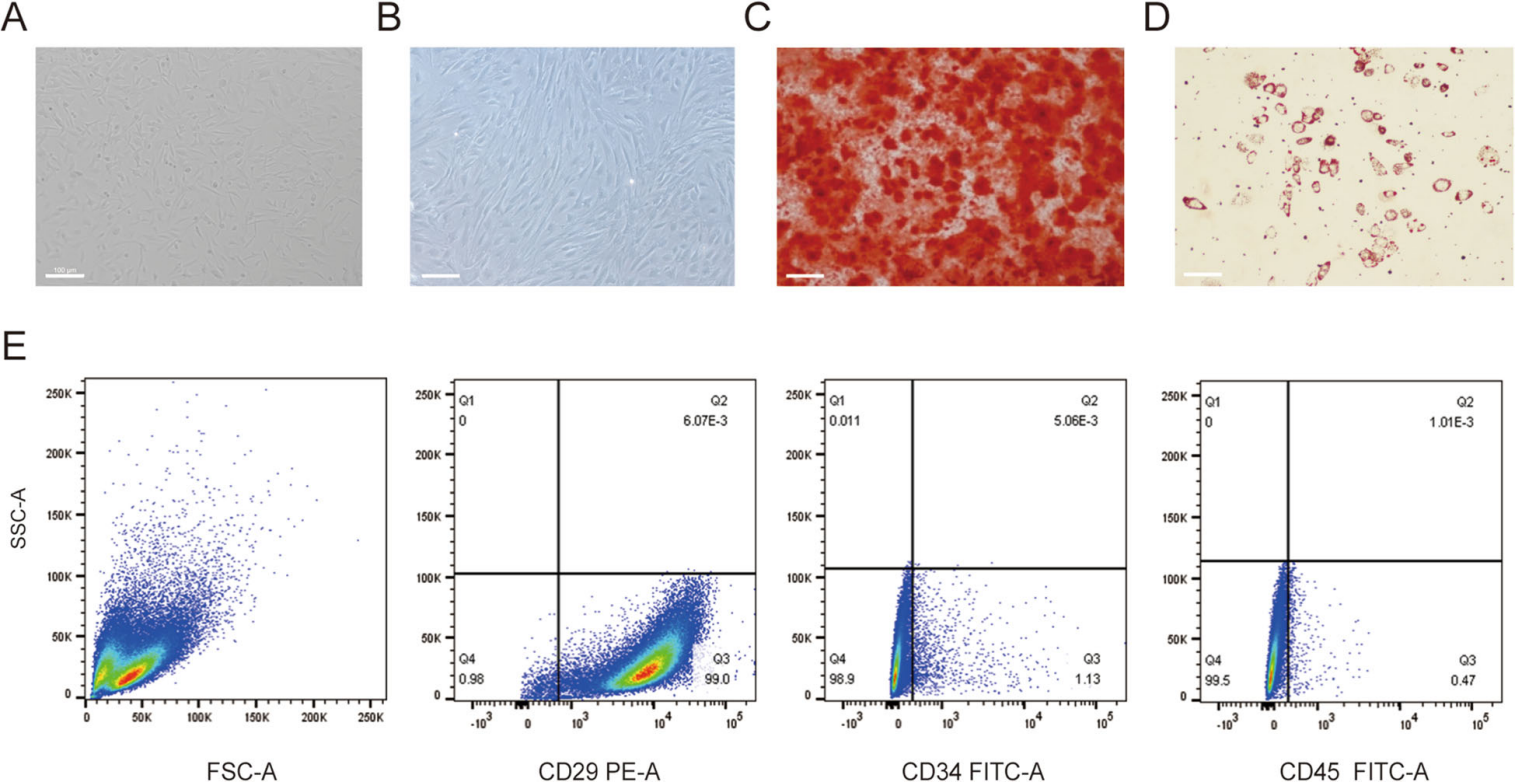
BMSC culture and characterization. **A** Passage 0 BMSCs. **B** Passage 3 BMSCs. **C** Passage 3 BMSCs stained with Alizarin Red after osteogenic differentiation. **D** Passage 3 BMSCs stained with Oil Red O after adipogenic differentiation. **D** Flow cytometry analysis of surface markers on passage 3 BMSCs. The percentage of cells positive for CD29 expression was greater than 95%, while that of cells positive for CD34 and CD45 was less than 2%

Morphological analyses of BMSCs isolated from aged osteoporotic rats (passages 0 and 3) revealed uniform and long spindle-shaped cells (Fig. 3A, B). After the induction of osteogenic differentiation, red calcium nodules were detected (Fig. 3C). After the induction of adipogenic differentiation, red lipid droplets were observed (Fig. 3D). The expression of BMSC (passage 3) surface markers was analysed, revealing a CD29 positivity rate greater than 95% and CD34 and CD45 positivity rates less than 2%, indicating that the cells were pure BMSCs (Fig. 3E).

**Fig. 4:**
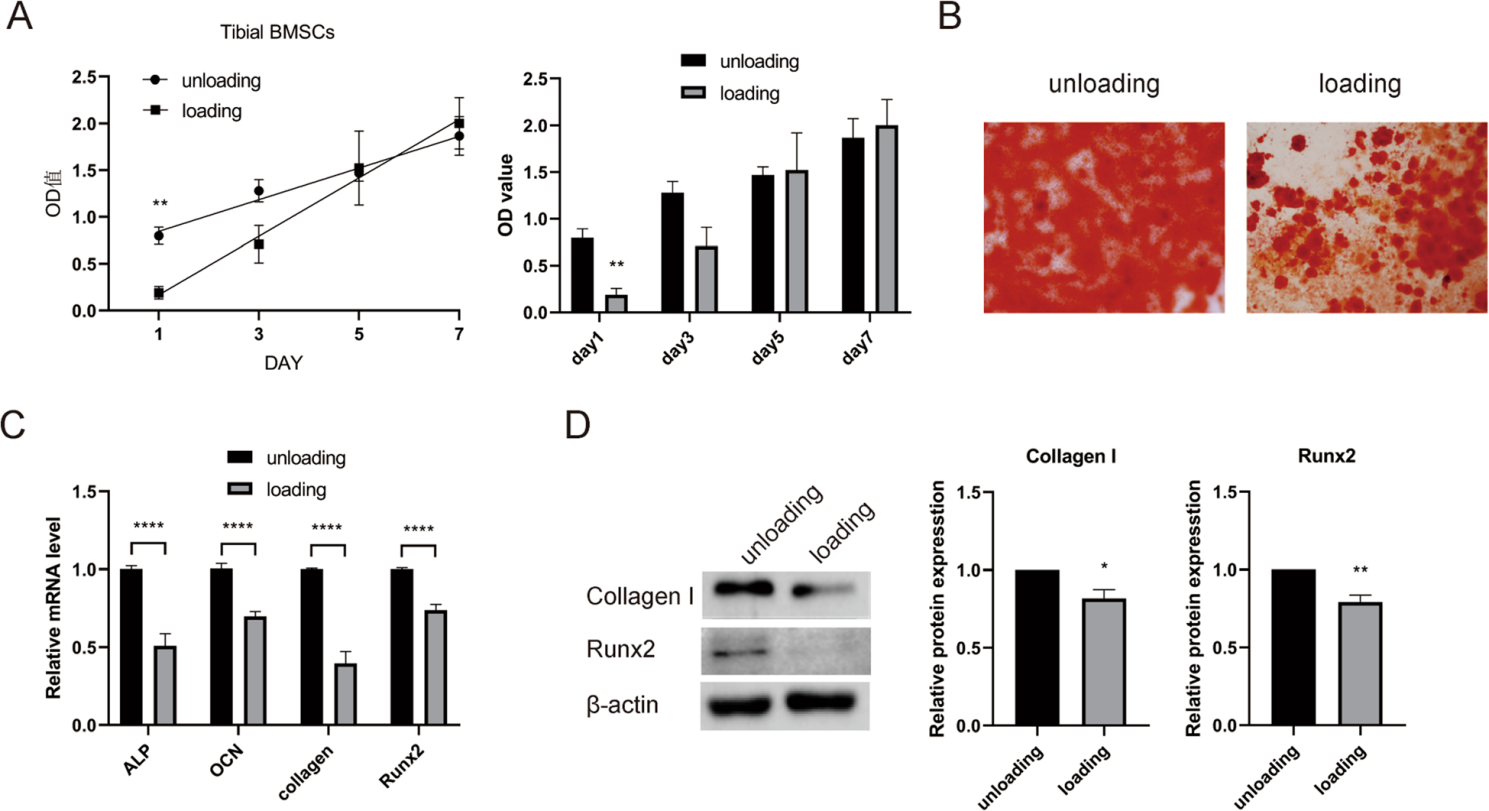
Comparison of BMSC proliferation and osteogenic differentiation between aged osteoporotic rats in the loading and unloading groups. **A** Comparison of proliferation ability at each time point; the proliferation ability of the loading group was lower than that of the unloading group on the first day, and the ability then gradually increased until there was no significant difference. **B** Osteogenic induction of BMSCs was performed for 14 days. Representative images of Alizarin Red staining show the widespread distribution of calcium salt deposits (red). **C** Compared with those in the unloading group, the mRNA expression levels of *ALP*, *OCN*, *Collagen I*, and *RUNX2* in the BMSCs of rats in the loading group were significantly lower than those in the unloading group. **D** The BMSC expression levels of collagen I and RUNX2 in the loading group were lower than those in the unloading group. The data are expressed as the mean ± SEM. **P < 0.01, ****P < 0.0001 vs. the unloading group

The proliferation ability of BMSCs was decreased in the loading group compared with the unloading group, with the difference on the first day being significant (*P* < 0.01), but the indifference was gradually ameliorated on the third, fifth, and seventh days (*P* > 0.05) (Fig. 4A). Alizarin Red staining showed decreased calcium node formation in the loading group (Fig. 4B). Compared with those in the unloading group, the levels of osteogenic genes, including *ALP*, *OCN*, *Collagen I*, and *RUNX2*, were reduced in the loading group (*P* < 0.001) (Fig. 4C), and the protein levels of Collagen I and Runx2 were similarly reduced (*P* < 0.05, *P* < 0.01) (Fig. 4D). These results suggest that the proliferation and osteogenic differentiation abilities of BMSCs isolated from aged osteoporotic rats were decreased after fatigue loading.

### Serum exosomes of young rats were internalized by the BMSCs of aged rats in the loading group to promote osteogenic differentiation

**Fig. 5:**
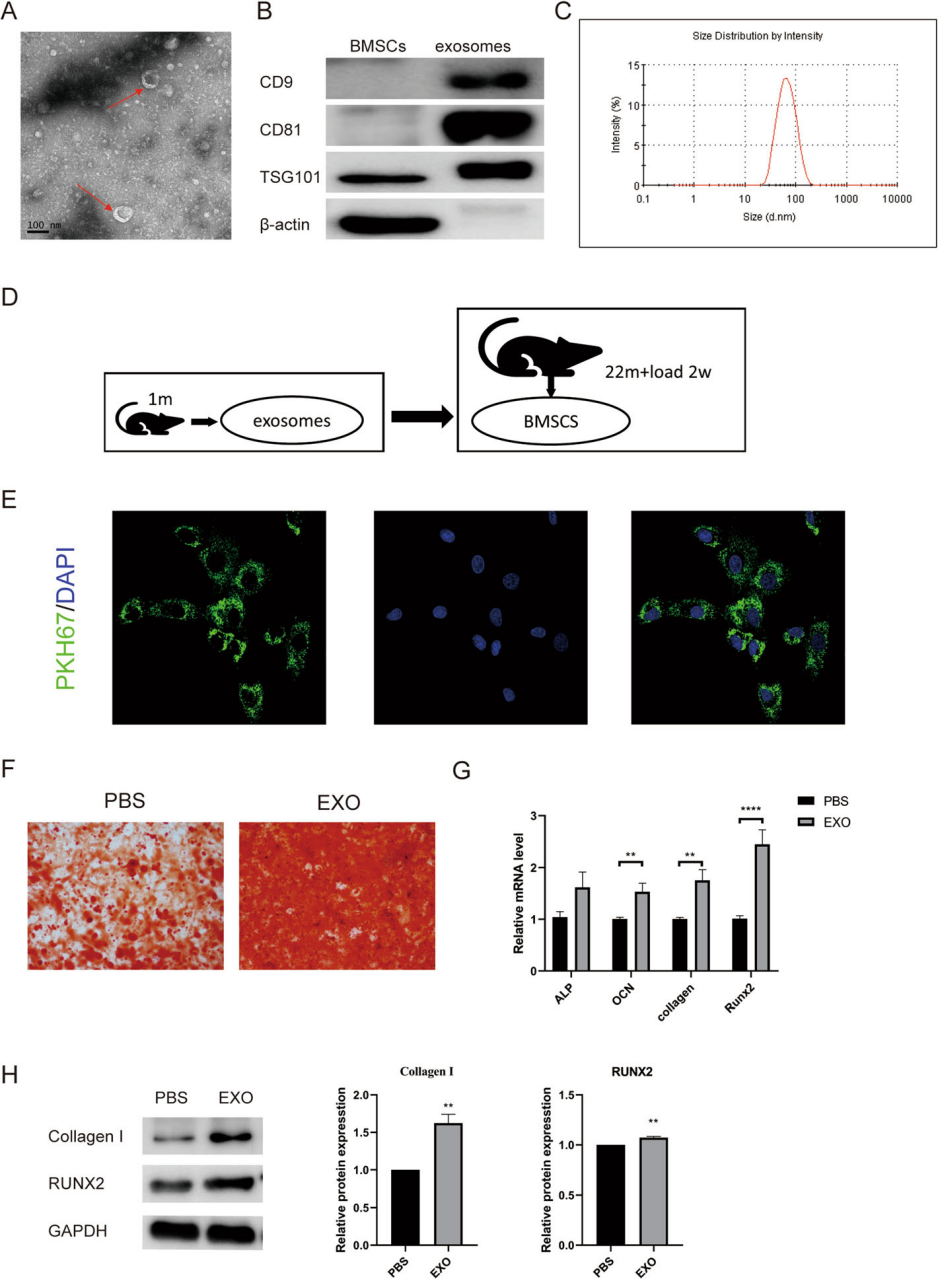
BMSCs promote the osteogenic differentiation of BMSCs in aged osteoporotic rats after fatigue loading. **A** Saucer-like structures of exosomes observed by transmission electron microscopy (TEM). Scale bar, 100 nm. **B** Positive exosomal marker proteins, CD9, CD81, and TSG101, in exosome-enriched preparations as detected by WB. **C** Particle size distribution of serum exosomes as analysed by DLS. **D** Illustration of BMSCs in bone microdamage model rats induced by serum exosomes from young rats. **E** Confocal fluorescence images of BMSCs and exosomes. **F** Representative images of the Alizarin Red staining of BMSCs treated with serum exosomes or PBS (control). **G** mRNA expression levels of *ALP*, *OCN*, *Collagen I*, and *Runx2* in BMSCs treated with serum exosomes or PBS. (H) Levels of collagen I and Runx2 in BMSCs treated with serum exosomes or PBS as detected by WB. The data are expressed as the mean ± SEM. *P < 0.05, **P < 0.01, ****P < 0.0001 vs. the PBS group (*n* = 3)

The serum exosomes of 1-month-old rats exhibited saucer-like structures with a diameter of ~ 100 nm as characterized by electron microscopy (Fig. 5A), and exosomal marker proteins, such as CD9, CD81, and TSG101, were detected in the serum exosomes (Fig. 5B). The particle size distribution of serum exosomes (Fig. 5C) showed a single peak with a diameter of approximately 71.35 nm, which is consistent with the characteristics of exosomes as determined by dynamic light scattering (DLS). BMSCs from aged rats in the loading group were treated with serum exosomes from young rats (EXO group) (Fig. 5D), and confocal fluorescence imaging of BMSCs and exosomes indicated many green fluorescent particles around the nuclei of BMSCs treated with exosomes, suggesting that the BMSCs internalized the serum exosomes (Fig. 5E). Alizarin Red staining showed greater calcium node formation in the EXO group than in the PBS group (Fig. 5F). The levels of the osteogenic genes *OCN*, *Collagen I*, and *Runx2* (*P* < 0.01, *P* < 0.01, *P* < 0.001) (Fig. 5G) and the protein levels of Collagen I and Runx2 were increased significantly in the EXO group compared with the PBS group (*P* < 0.01) (Fig. 5H). These results suggested that osteogenic differentiation was promoted in BMSCs treated with serum exosomes derived from young rats.

### miRNA-19b-3p promoted the osteogenic differentiation of BMSCs

**Fig. 6:**
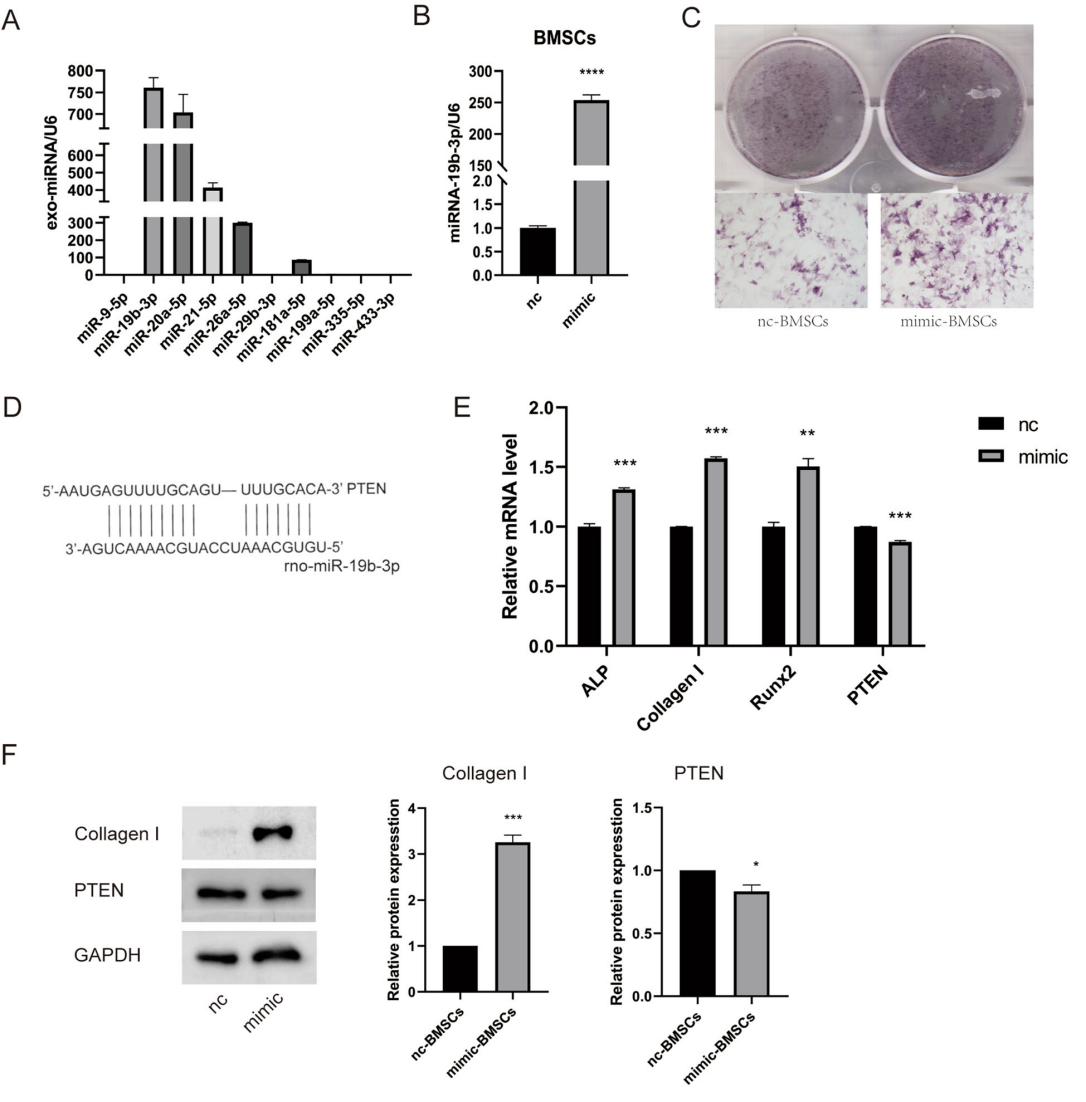
miRNA-19b-3p promoted the osteogenic differentiation of BMSCs. **A** Expression of 10 miRNAs related to osteogenesis in serum exosomes from young rats; high expression of miR-19b-3p, miR-20a-5p, miR-21-5p, and miR-26b-5p was detected. **B** Transfection efficiency of the miR-19b-3p mimic. **C** BMSCs were stained for ALP after induction. **D** Conserved seed sequence of miR-19b-3p in the 3′UTR of PTEN mRNA. **E** After miRNA-19b-3p mimic transfected, the mRNA expression levels of *ALP*, *Collagen I*, and *RUNX2* in BMSCs were increased, whereas that of the predicted gene *PTEN* was decreased. **F** WB analysis of the levels of PTEN in BMSCs treated with the miR-19b-3p mimic and nc. The BMSCs of the mimic group expressed higher levels of Collagen I and lower levels of PTEN. The data are expressed as the mean ± SEM (*n* = 3). *P < 0.05, **P < 0.01, ****P < 0.0001

Among miRNAs related to osteogenesis, we detected 10 in the serum exosomes of young rats and found that the levels of miRNA-19b-3p, miR-20a-5p, miR-21-5p, and miR-26b-5p were high and that the level of miRNA-19b-3p was significantly high (Fig. 6A).

To study the effect of miRNA-19b-3p on the osteogenic differentiation of BMSCs, we transfected BMSCs with miRNA-19b-3p mimic or negative control. After treating BMSCs with miRNA-19b-3p mimic to upregulate miRNA-19b-3p, the transfection efficiency of the miRNA-19b-3p mimic was detected by RT-qPCR, revealing that the miRNA-19b-3p expression was significantly increased (*P* < 0.001) (Fig. 6B). After osteogenic induction, the ALP staining of BMSCs transfected with the miRNA-19b-3p mimic (mimic group) was significantly deeper compared with that of BMSCs transfected with the negative control (nc group) (Fig. 6C). The MicroRNA Target Prediction Database **(**miRDB) showed the possible target protein PTEN and the binding site of rno-miR-19b-3p in the 3′UTR of PTEN (Fig. 6D). The levels of osteogenic genes, including *ALP*, and *Collagen I*, *RUNX2* were increased (*P <* 0.001, *P <* 0.01, *P <* 0.01), The level of the gene *PTEN* was decreased in the mimic group compared with the nc group (*P <* 0.001) (Fig. 6E). The protein levels of Collagen I was increased (*P <* 0.001) and PTEN was decreased (*P <* 0.05) (Fig. 6F).

### miRNA-19b-3p in the serum exosomes of young rats promoted the osteogenic differentiation of BMSCs in aged rats after fatigue loading

**Fig. 7:**
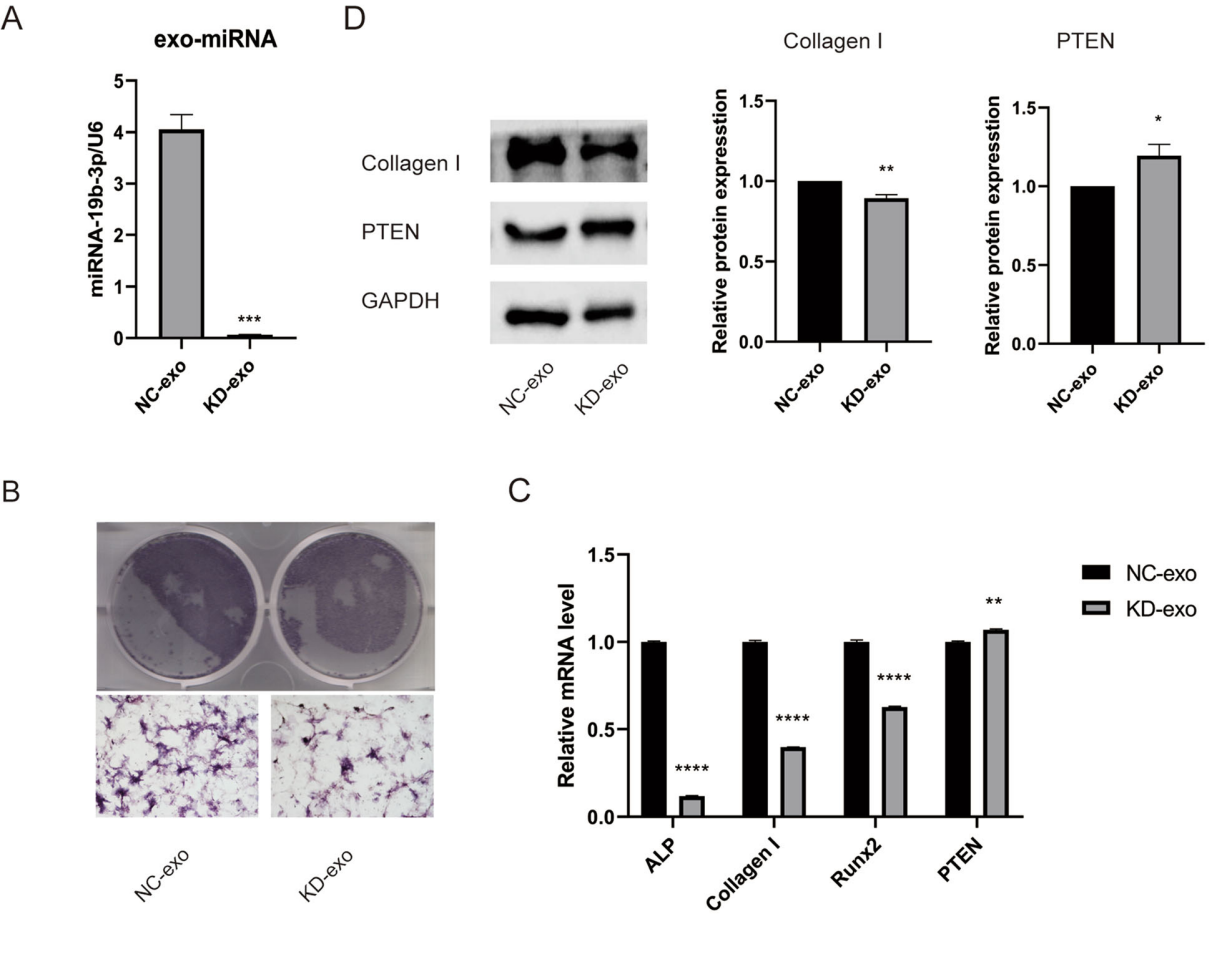
The promotion of osteogenic differentiation by serum exosomes from young rats is partly mediated by miR-19b-3p. **A** Transfection efficiency of the miR-19b-3p inhibitor. **B** Under osteogenic induction, BMSCs treated with exosomes with miRNA-19b-3p knockdown BMSCs were stained for ALP after induction. **C** After miRNA-19b-3p knockdown in exosomes, the mRNA expression levels of *ALP*, *Collagen I*, and *RUNX2* in BMSCs were reduced, whereas that of the predicted gene *PTEN* was increased. **D** WB analysis of the levels of PTEN in BMSCs treated with the NC-exo and KD-exo. The BMSCs of the KD-exo group expressed lower levels of Collagen I and higher levels of PTEN. The data are expressed as the mean ± SEM (*n* = 3). *P < 0.05, **P < 0.01, ****P < 0.0001

After treating young serum exosomes with miRNA-19b-3p inhibitor to downregulate miRNA-19b-3p, the transfection efficiency of the miRNA-19b-3p inhibitor was detected by RT-qPCR, revealing that the miRNA-19b-3p expression in the exosomes was significantly reduced (*P* < 0.001) (Fig. 7A). The ALP staining of BMSCs derived from exosomes transfected with the miRNA-19b-3p inhibitor (KD-exo group) was significantly lighter compared with that of BMSCs derived from exosomes transfected with the negative control (NC-exo group) (Fig. 7B). The levels of osteogenic genes, including *ALP*, *Collagen I* and *RUNX2* were decreased in the KD-exo group compared with the NC-exo group (*P <* 0.01, *P <* 0.001*, P <* 0.05), the level of *PTEN* was increased in the KD-exo group compared with the NC-exo group (*P <* 0.05) (Fig. 7C). The protein levels of Collagen I were decreased (*P <* 0.01), PTEN was increased (*P <* 0.05) (Fig. 7D). These results suggest that the knockdown of miRNA-19b-3p in exosomes reduced their promotional effect on BMSCs possibly by targeting PTEN.

## Discussion

In this study, we performed OVX in 7-month-old female SD rats to establish an osteoporotic rat model. While the BMD was decreased after OVX, it did not continue to decrease for 22 months. Postmenopausal bone loss is accelerated, and the BMD decreases significantly and then decreases slowly. Clinical studies have found that the BMD is relatively stable in humans at 70–80 years old; however, the prevalence of brittle fractures increases over this period [25]. Bone quality is associated with not only the BMD but also microdamage accumulation, properties of the collagen/mineral matrix, and other factors [26], ultimately resulting in discordance between changes in brittle fractures and bone density in elderly patients [25]. Thus, the BMD cannot be monitored, and the measurement of other factors, including microdamage, is necessary for the accurate evaluation of bone quality in the elderly population.

Bone microdamage mainly includes linear microcracks and diffuse damage, the former of which is more common [27]. Linear microcracks refer to small fractures ranging in size from 50 to 100 μm with a sharp contour on the bone cross-section. A previous study demonstrated that old-donor bone (83 ± 3 years) contained more linear microcracks in the cortices, whereas young-donor bone (40 ± 10 years) contained more diffuse damage than older donor bone [28]. The accumulation of linear microcracks is related to decreased bone mechanical properties, such as hardness, strength, and toughness [26]. We found more microcracks in the tibias of aged rats with osteoporosis subjected to fatigue loading than in control rats, although the bone microstructure and biomechanical properties were not significantly different after loading for two weeks. This indicates that the microstructure and mechanical properties of the bone did not change during the early stage of microdamage and were affected only when microdamage accumulated, suggesting the importance of early bone microdamage repair.

The rapid adaptation of bone to changes in its mechanical environment to ensure the proper placement of a sufficient bone mass to withstand functional areas of activity has been described as Wolff’s law [29]. Losing mechanical load leads to bone loss [30, 31], and a certain load can prevent bone loss due to disability and bone functional waste [32]. The mechanical load is also closely related to the rate and mass of bone formation [33–35], and high levels of physical activity are associated with a higher bone mass and a lower fracture risk and are recommended for fracture prevention [36–41]. The mechanical strain has long been considered an important factor in bone remodelling, and numerous cell culture models have been proposed previously to study the effect of mechanical stimulation on cell differentiation [42]. Previous studies have studied the effects of mechanical stimulation on cell proliferation and differentiation in vitro. Mesenchymal stem cells (MSCs) are highly sensitive to the biomechanics of their extracellular environment [43], and mechanical loading can affect mesenchymal stem cells and determine their differentiation fate [44]. Moreover, the mechanical strain was used to improve bone marrow mesenchymal stem cell osteogenesis [45–49], and while the underlying mechanisms were still not fully clarified [50], the ERK1/2 [46], MAPK, and Wnt/β-catenin signalling pathways may play a role [51–53]. Studies have shown that the mechanical sensitivity of MSCs is related to age [54]. In this study, the BMSCs of ageing osteoporosis rats were less capable of osteogenic differentiation than those of unloaded rats after fatigue loading in vivo. These results suggested that BMSCs are not stimulated under fatigue loading in ageing osteoporosis rats, which can enhance osteogenic differentiation and participate in the repair of bone microdamage, thus providing a possible explanation for the decreased bone repair ability in elderly individuals. The impaired osteogenic capacity of BMSCs may be caused by mechanical signals, suggesting that bone quality cannot be improved by increasing mechanical stimulation at all ages. Sometimes, the osteogenic differentiation capacity of BMSCs in elderly individuals can be suppressed, thus aggravating microdamage accumulation and increasing fracture risk. Therefore, improving the osteogenic differentiation ability of aged BMSCs under mechanical loading may promote bone microdamage repair in aged rats. For the elderly population, external physical therapy for osteoporosis may not always have a positive effect.

Current treatments for osteoporosis rely on drugs, such as bisphosphonates, that prevent bone resorption but do not restore bone loss [55–57]. To date, there are fewer drugs used to induce bone formation, commonly used such as parathyroid hormone (PTH) (1–34) [58]. In addition, most of the current research mainly focuses on promoting healing after osteoporotic fracture, and few treatments are available for the repair of bone microdamage before osteoporosis fractures in the elderly population. We herein found that the reduced osteogenic differentiation ability of BMSCs was promoted by the use of serum exosomes derived from young rats to intervene with BMSCs derived from aged rats subjected to fatigue loading. This result indicates that the serum exosomes of young rats can stimulate the osteogenic differentiation of BMSCs in rats with obvious bone microdamage after fatigue loading. Exosomes can escape attack by the immune system, and young exosomes capable of promoting the repair of bone microdamage in the elderly serve as a new treatment idea for improving bone quality and preventing fractures in elderly patients with osteoporosis.

Exosomes contain bioactive molecules, such as proteins and miRNAs, that promote the osteogenic differentiation of BMSCs [59]. We detected high levels of osteogenic-related miRNAs, such as miRNA-19b-3p, miR-20a-5p, miR-21-5p, and miR-26a-5p, in the serum exosomes of young rats, suggesting that their promotional effect on osteogenic differentiation is related to these highly expressed miRNAs. Studies have shown that the plasma miRNA-19 levels in elderly people are lower than those in young people and that the level of miR-19b is related to the BMD [60]. Transfection with miRNA-19b-3p mimic can promote the osteogenic differentiation of hMSCs and MC3T3-E1 cells, and the injection of a chemically modified miRNA-19b (agomiR-19b) can reduce the osteoporotic bone phenotype in aged ovariectomized mice [60]. In our study, the osteogenic differentiation of BMSCs was enhanced by transfection with miRNA-19b-3p mimic, which confirmed the effect of miR-19b-3p on osteogenic differentiation in the BMSCs. These are consistent with previous literature reports. Moreover, our results show that the osteogenic differentiation of BMSCs was enhanced transfected with the miRNA-19b-3p mimic, which is consistent with previous literature reports. The osteogenic effect of exosomes transfected with the miRNA-19b-3p inhibitor was decreased compared with that of the NC-exo group, confirming that serum exosomes derived from young rats can improve the osteogenic differentiation of BMSCs in aged OVX rats with bone microdamage partly via the action of miRNA-19b-3p.

The MicroRNA Target Prediction Database (miRDB) showed the possible target protein PTEN. The targeting effect of miRNA on PTEN has been demonstrated in previous literatur e[60–63]. Moreover, PTEN negatively regulates the differentiation of osteoblasts [64–66] Thus, we further tested the gene and protein levels of PTEN. Our research shows that under osteogenic induction, the PTEN expression in the BMSCs of aged rats under fatigue loading subjected to treatment with miRNA-19b mimics was decreased, whereas it was increased after exosome intervention and transfection of the miR-19b-3p inhibitor. These results indicate that the miRNA-19b-3p achieve this effect possibly by regulating PTEN.

The limitations of this study include the lack of exosome targeting and the rarity of elderly rat samples, which together contributed to the failure to verify the effects of serum exosomes from young rats on the microdamage repair, new bone formation, bone microstructure, and bone biomechanical properties of elderly osteoporotic rats in vivo. Future studies should consider the use of an adaptor [67] to increase the bone targeting of serum exosomes and precisely act on bone tissues and BMSCs to confirm their effect.

## Conclusions

This study illustrates that the osteogenic differentiation of BMSCs in aged osteoporotic rats is decreased after fatigue loading, and the decrease can be improved by serum exosomes derived from young rats, partly via miRNA-19b-3p. This result suggests that bone quality cannot be improved at all age stages by increasing mechanical stimulation, and age should thus potentially be considered during mechanical treatment. Serum exosomes from young rats may help to develop new treatment strategies and prevent fractures in aged patients with osteoporosis and bone microdamage.

## Supplementary Information

**Additional file 1: Figure S1.** Electronic fatigue damage machine to induce fatigue loading in aged osteoporotic rats. (**A**) An electronic fatigue damage machine was used to complete the four-point bending fatigue test. (**B**) Force-bearing points and the distance. (**C**) Graphic illustration of the force-bearing points and distance. (**D**) The region of interest (ROI) selected for analysis. The X position was assigned based on the load point, and a region encompassing a minimum distance of 3–5 mm and a maximum distance of 8–10 mm from the X position was selected as the region of interest (ROI) for analysis.

## Data Availability

The authors confirm that all data and materials underlying the findings are fully available without restriction. All relevant data and materials are within the paper and its Supporting Information files.

## Abbreviations

BMSC: Bone mesenchyme stem cell
PTEN: Phosphatase and tensin homologue deleted from chromosome 10
TEM: Transmission electron microscope
NTA: Nanoparticle tracking analysis
WB: Western blotting
RT-qPCR: Quantitative real-time PCR
DXA: Dual-energy X-ray absorptiometry
OVX: Ovariectomy
EVs: Extracellular vesicles
BMD: Bone mass or bone density
miRDB: MicroRNA Target Prediction Database
