# Supplementary figures and images for: Serum exosomes from young rats improve the reduced osteogenic differentiation of BMSCs in aged rats with osteoporosis after fatigue loading in vivo
Source: Stem Cell Res Ther. 2021 Jul 27;12:424. doi: 10.1186/s13287-021-02449-9 (PMC8314589; doi:10.1186/s13287-021-02449-9)

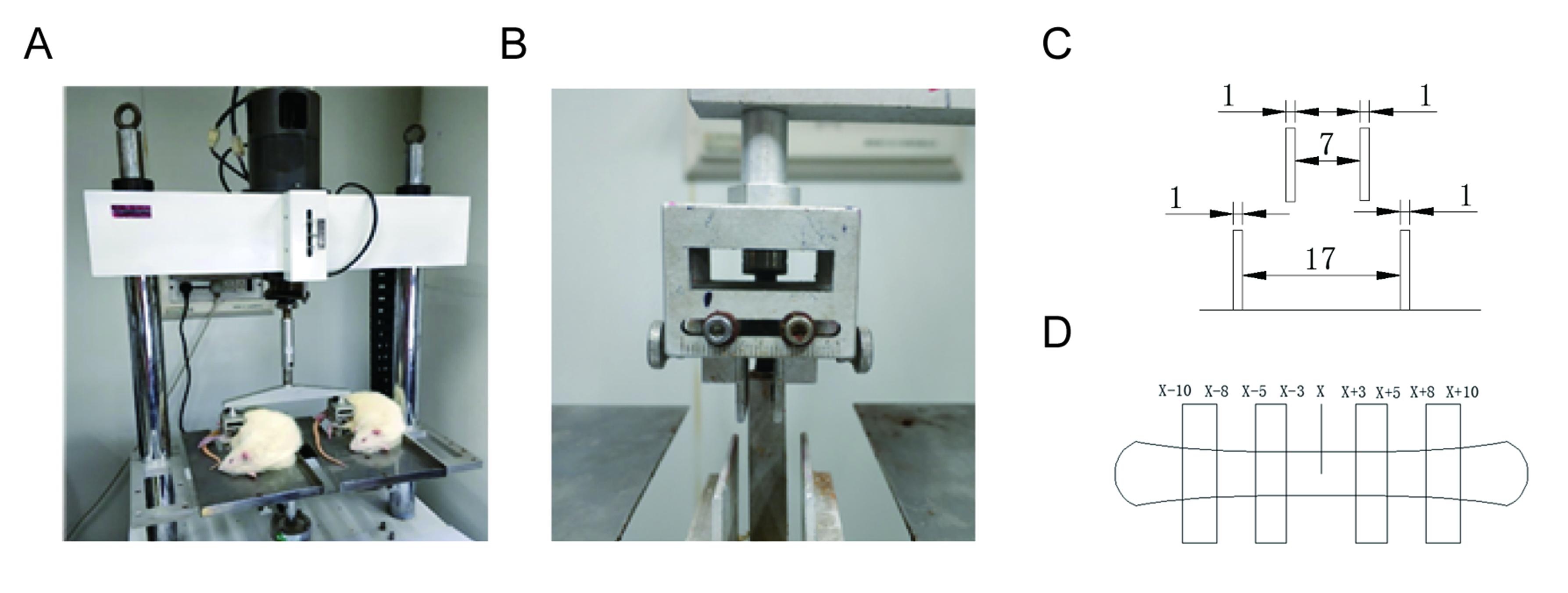

Supplement: Supplementary file 1 — Additional file 1: Figure S1. Electronic fatigue damage machine to induce fatigue loading in aged osteoporotic rats. (A) An electronic fatigue damage machine was used to complete the four-point bending fatigue test. (B) Force-bearing points and the distance. (C) Graphic illustration of the force-bearing points and distance. (D) The region of interest (ROI) selected for analysis. The X position was assigned based on the load point, and a region encompassing a minimum distance of 3–5 mm and a maximum distance of 8–10 mm from the X position was selected as the region of interest (ROI) for analysis. [file 13287_2021_2449_MOESM1_ESM.tif]
